# Inspiratory-expiratory variation of pleural line thickness in neonates with and without acute respiratory failure

**DOI:** 10.1186/s12931-023-02651-8

**Published:** 2024-01-04

**Authors:** Barbara Loi, Pasquale Fabio Barra, Laura Vivalda, Francesco Raimondi, Daniele De Luca

**Affiliations:** 1 Division of Pediatrics and Neonatal Critical Care, “A.Béclère” Medical Center, Paris- Saclay University Hospitals, APHP, Paris, France; 2 Physiopathology and Therapeutic Innovation Unit-INSERM U999, Paris-Saclay University, Paris, France; 3 Division of Neonatology, Department of Translational Medical Sciences, Università “Federico II” di Napoli, Naples, Italy

**Keywords:** Pleura, Infants, Respiratory distress, Spontaneous breathing, Ultrasound

## Abstract

**Background:**

There are relatively few data about the ultrasound evaluation of pleural line in patients with respiratory failure. We measured the pleural line thickness during different phases of the respiratory cycle in neonates with and without acute respiratory failure as we hypothesized that this can significantly change.

**Methods:**

Prospective, observational, cohort study performed in an academic tertiary neonatal intensive care unit recruiting neonates with transient tachypnoea of the neonate (TTN), respiratory distress syndrome (RDS) or neonatal acute respiratory distress syndrome (NARDS). Neonates with no lung disease (NLD) were also recruited as controls. Pleural line thickness was measured with high-frequency ultrasound at end-inspiration and end-expiration by two different raters.

**Results:**

Pleural line thickness was slightly but significantly higher at end-expiration (0.53 [0.43–0.63] mm) than at end-inspiration (0.5 [0.4–0.6] mm; *p* = 0.001) for the whole population. End-inspiratory (NLD: 0.45 [0.38–0.53], TTN: 0.49 [0.43–0.59], RDS: 0.53 [0.41–0.62], NARDS: 0.6 [0.5–0.7] mm) and -expiratory (NLD: 0.47 [0.42–0.56], TTN: 0.48 [0.43–0.61], RDS: 0.53 [0.46–0.65], NARDS: 0.61 [0.54–0.72] mm) thickness were significantly different (overall *p* = 0.021 for both), between the groups although the absolute differences were small. The inter-rater agreement was optimal (ICC: 0.95 (0.94–0.96)). Coefficient of variation was 2.8% and 2.5% for end-inspiratory and end-expiratory measurements, respectively. These findings provide normative data of pleural line thickness for the most common forms of neonatal acute respiratory failure and are useful to design future studies to investigate possible clinical applications.

**Supplementary Information:**

The online version contains supplementary material available at 10.1186/s12931-023-02651-8.

Lung ultrasound provides useful information about the aeration of lung tissue, but also allows an easy visualization of most of the pleura (with the exception of the paravertebral and diaphragmatic areas) [1]. The pleura is usually 0.3–0.4 mm thick, however the thickness of the ultrasound-detected pleural line may be increased by the movements of pleural sheets during the respiratory cycle [1]. Thus, in healthy adults, the pleural line thickness can reach approximately 1 or 2 mm, if the measurements are realized with linear or convex probes, respectively [2]. Pleural line thickness is further increased in patients with lung diseases and may show several qualitative anomalies [3].

In neonatology, despite point-of-care lung ultrasound is increasingly being use for its advantages (e.g.: accuracy, quickness, ease and reduced invasiveness), [4] the ultrasound evaluation of pleura has received little attention so far. In a recent preliminary study, the normal pleural line thickness was slightly below or above 1 mm, if measured with micro-linear or -convex probes, respectively [5]. Nonetheless, the thickness has never been studied during different phases of the respiratory cycle in patients with various types of acute respiratory failure. This is important to allow a detailed description of pleural line anomalies and the ultrasound characterization of neonatal respiratory disorders. We aimed to fill this gap and we hypothesized that pleural line thickness can change during the respiratory cycle and in different forms of neonatal acute respiratory failure.

This was a prospective, observational, cohort study performed in an academic tertiary neonatal intensive care unit (NICU). As lung ultrasound is our first-line imaging technique, [6] the study was embedded in routine clinical care which was not changed for study purposes. Local ethical approval was granted, parental informed consent was obtained upon NICU admission and all relevant privacy regulations were respected. Patients were eligible if they were admitted to the NICU within the first 72 h of life and diagnosed with transient tachypnoea of the neonate (TTN), [7] respiratory distress syndrome (RDS) due to primary surfactant deficiency, [7] or neonatal acute respiratory distress syndrome (NARDS) [8]. These disorders were diagnosed using consensus criteria based on the integration of clinical and imaging data [7, 8]. Lung ultrasound was used as imaging tool and interpreted according to international guidelines, [9] based on classical semiology [4]. Patients with no lung disease (NLD) were also eligible as controls if they were NICU-admitted within the first 72 h of life for non-respiratory reasons, did not need supplemental oxygen and presented with normal lung ultrasound and chest clinical examination. Exclusion criteria were major congenital malformations, chromosomopathies, congenital lung anomalies, thoracic surgery and airleaks or pleural effusions.

Clinical data of enrolled patients were anonymously recorded in real-time. The same investigator (PLB) performed all exams, upon NICU admission and within the first 72 h of life, on patients lying supine, during routine clinical care, before surfactant administration, if any. A micro-linear, hockey-stick, high-frequency (15 MHz) probe (CX-50®, Philips, Eindhoven, Netherlands) was used with the focus set over the pleural line and gain optimized to make it thinnest. Further settings were as previously described [10]. High definition clips (600 dpi,30”) were recorded during the exams on the right upper anterior quadrant from two (one transversal and one longitudinal) scans, avoiding consolidated lung zones (where the pleural line is interrupted). This quadrant was chosen for accessibility and to standardize the measurement. Clips were then played slowly and reviewed off-line: end-inspiration and end-expiration were identified, and the pleural line thickness was measured, on a single breath per each videoclip, using the internal software of the ultrasound machine. Measurements were done for each respiratory phase, on selected frames where the pleural line was parallel to the probe without image magnification. The values obtained from transversal and longitudinal scans were averaged for each of the two respiratory phases. For confirmation, these offline reviews and measurements were also performed by an independent investigator (BL) blinded to the previous measurements. The maximal axial resolution for the measurement was 0.01 mm.

Pleural line thickness was expressed as median [25th -75th percentile] and compared with Wilcoxon or Kruskal-Wallis test, followed by Conover-Iman *post hoc* test, as appropriate. Intraclass correlation coefficient (ICC, 95% confidence interval) was used to evaluate the inter-rater absolute agreement. Mann-Withney test and Spearman correlation were used to study if pleural thickness varied with dichotomous perinatal characteristics or was correlated with continuous demographic or clinical variables. Ours was the first work measuring the ultrasound-assessed pleural line thickness during different phases of the respiratory cycle, therefore, to estimate the repeatability of measurements, the intra-rater coefficient of variation (CV) was calculated over the separate measurements for each observer. A CV of less than 10% was considered as low data dispersion (i.e.: good repeatability), as previously published [11]. As this was the first study of its kind, a formal sample size calculation was unfeasible and we choose a convenience sample size of 100 neonates, which was larger than the unique preliminary neonatal study available [5]. Analyses were performed with JASP (v.0.17.1; JASP Team (2023)) and *p* < 0.05 were considered significant.

Table 1 describes basic characteristics of the enrolled population. The online supplement shows two illustrative videoclips (one longitudinal and one transversal) used for the measurements. Figure 1 shows illustrative still frames taken from the videoclips with markers to measure the end-expiratory thickness. Pleural line thickness was slightly but significantly higher at end-expiration (0.53 [0.43–0.63] mm) than at end-inspiration (0.5 [0.4–0.6] mm; *p* = 0.001; Fig. 2**A**) for the whole population, and only two measurements resulted above 1 mm. Subgroup analysis showed the end-expiration to be higher than the end-inspiration thickness in NLD (0.47 [0.42–0.56] vs. 0.45 [0.38–0.53] mm; *p* = 0.049) and NARDS (0.61 [0.54–0.72] vs. 0.6 [0.5–0.7] mm; *p* = 0.04) patients. Figure 2**B-C** shows data for patient subgroups: both inspiratory (NLD: 0.45 [0.38–0.53], TTN: 0.49 [0.43–0.59], RDS: 0.53 [0.41–0.62], NARDS: 0.6 [0.5–0.7] mm) and expiratory (NLD: 0.47 [0.42–0.56], TTN: 0.48 [0.43–0.61], RDS: 0.53 [0.46–0.65], NARDS: 0.61 [0.54–0.72] mm) thickness were different between the groups (overall *p* = 0.021, for both inspiration and expiration) while raincloud plots show a partial data overlap; significant *post hoc* comparisons show that RDS and NARDS patients had significantly thicker pleural line compared to controls. The agreement between the two raters was optimal (ICC: 0.95 (0.94–0.96)) and the CV was 2.8% (on average 0.014 mm) and 2.5% (on average 0.013 mm) for inspiratory and expiratory measurements, respectively. Pleural thickness was similar in neonates of different sex or perinatal characteristics; there was no significant correlation between thickness and gestational age, birth weight, Apgar score or mean airway pressure (data not shown).

Our findings demonstrate that the pleural line thickness in neonates is usually below 1 mm, with a slight but noticeable difference between end-inspiration and -expiration, due to the volume of the underlying lung that is changing during the respiratory cycle. This difference is statistically significant but not clinically meaningful, given the overlap of data between patients with different types of acute respiratory failure. Thus, the pleural line thickness may not be helpful for the differential diagnosis of neonatal respiratory failure and this is consistent with data accumulated in adults and in smaller preliminary neonatal studies [3, 5]. Nonetheless, patients with more severe respiratory disorders (i.e.: RDS and NARDS) have a thicker pleural line compared to controls. This seems coherent since lung ultrasound findings in these cases often include pleural line anomalies and subpleural consolidations [4]. Our findings are also consistent with those of a earlier preliminary work, which, however, reported a higher thickness in RDS patients [5]. This may be due to subpleural consolidations typical of this disease that may have been considered in the thickness measurement.

Given the very low CV, [11] this work also demonstrates the repeatability of pleural thickness measurement using high-frequency ultrasound. This can be interesting as these probes have higher superficial resolution and can be used for the same purpose in adults too. It remains to be established if ultra-high frequency probes can further improve the measurement [12]. It is conceivable that the inspiratory-expiratory difference could have a role in the detection of pneumothorax (where the lung sliding is abolished) or the evaluation of lung overdistention but these are interesting hypotheses to be verified in dedicated studies. The relationship between the inspiratory-expiratory difference and the diaphragmatic activity also deserves to be investigated. We acknowledge some study limitations. First, we only performed measurements in the right upper chests: although never been demonstrated so far, one could conceive that lung aeration, differing between lung zones and levels of respiratory support, could influence the change in pleural line thickness. Thus, by exploring more chest zones, we might have detected more differences in pleural line thickness. The severity of respiratory failure in our population was relatively moderate, so results cannot be immediately translated to more severe patients, although these would obviously present with consolidated lung zones where pleural line is interrupted and not measurable.

Finally, we have a relatively small sample size for subgroups of neonates with different respiratory disorders (particularly for those who are not affected by RDS), thus the subgroup analysis might be underpowered. In conclusion, ultrasound-assessed pleural thickness in neonates is between 0.3 and 1 mm with a significant difference between inspiration and expiration. Thickness is greater in patients with RDS and NARDS than in controls without respiratory failure. This helps to design future studies focusing on clinical application of the inspiratory-expiratory difference.

**Table 1:** Basic population details

	Whole population (124)	RDS (69)	TTN (14)	NARDS (15)	NLD (26)
Gestational age (weeks)	31.9 (3.9)	29.9 (3.2)	35.2 (3)	33 [2.8]	35 (3.6)
Birth weight (g)	1700 (836)	1301 (580)	2621 (779)	1796 (686)	2209 (871)
Males	67 (54%)	36 (52.2%)	10 (71.4%)	8 (53.3%)	13 (50%)
Cesarean section	70 (56.5%)	45 (65.2%)	6 (42.9%)	6 (40%)	13 (50%)
Prenatal steroids	77 (62.1%)	50 (72.5%)	5 (35.7%)	9 (60%)	13 (50%)
Clinical chorioamnionitis	24 (19.3%)	19 (27.5%)	1 (7.1%)	2 (13.3%)	2 (7.7%)
5’ Apgar score	9 [8–10]	9 [8–10]	8 [7–9]	8 [7–10]	9 [8–10]
CRIB-II score	6 [2–9]	8 [4–9]	1 [0–1]	2 [2–5]	3 [0–4]
SNAPPE-II score	0 [0–12]	0 [0–7]	0 [0–4]	16 [6–28]	0 [0–18]
Respiratory support					
None	24 (19.3%)	0	0	0	24 (92.3%)
Non-invasive	90 (72.6%)	65 (94.2%)	14 (100%)	11 (73.3%)	0
Invasive	10 (8.1%)	4 (5.8%)	0	4 (26.7%)	2 (7.7%)

Data are expressed as mean (standard deviation), median [25th – 75th percentile] or number (%). Prenatal steroids were considered when two 12 mg betamethasone doses were administered at least 24 h before the delivery. CRIB-II and SNAPPE-II scores were calculated for neonates ≤ or > 32 weeks’ gestation, respectively. Respiratory support was considered as non-invasive (i.e.: continuous positive airway pressure or non-invasive ventilation) or invasive (i.e.: conventional or high-frequency oscillatory ventilation). Abbreviations: CRIB: critical risk index for babies; NARDS: neonatal acute respiratory distress syndrome; NLD: no lung disease; Paw: mean airway pressure; RDS: respiratory distress syndrome; SNAPPE: score for neonatal acute physiology-perinatal extension; TTN: transient tachypnoea of the neonate

**Fig. 1:**
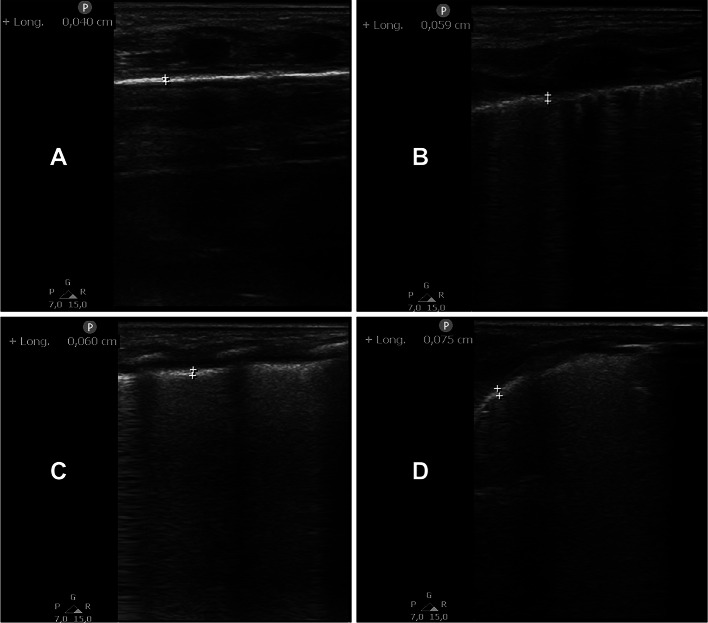
Illustrative pictures of ultrasound measurement of pleural line thickness. Pictures were end-expiration still frames extracted from the clips recorded during the exams and used for pleural line measurements. Measurements are shown by + symbols. Neonates with no lung disease (NLD, **A**), transient tachypnea of the neonate (TTN, **B**), respiratory distress syndrome due to primary surfactant deficiency (RDS, **C**) and neonatal acute respiratory distress syndrome (NARDS, **D**) are shown

**Fig. 2:**
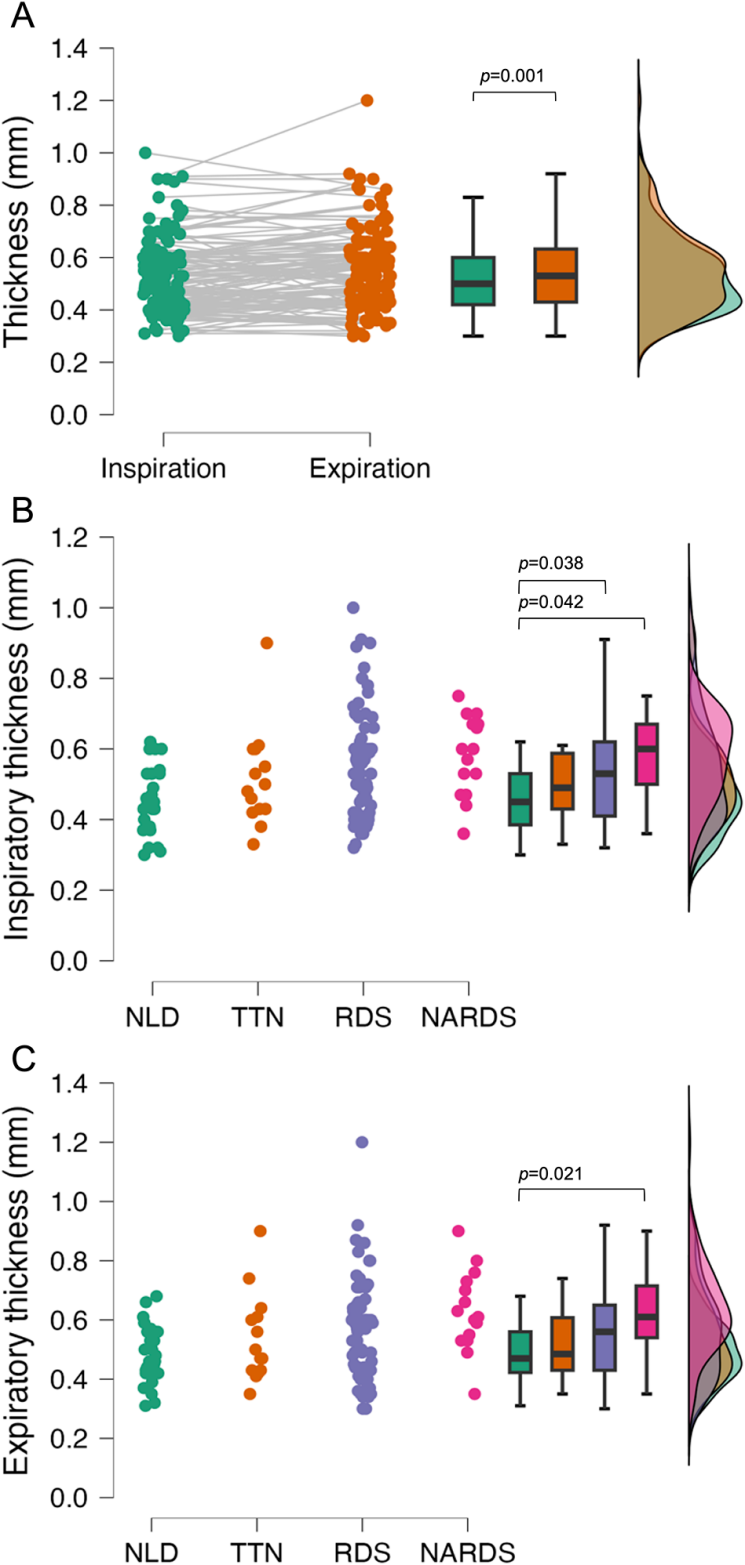
Pleural line thickness. **A** depicts data for the whole population (n = 124) during inspiration (green dots) and expiration (orange dots); data were compared with paired Wilcoxon test (*p* = 0.001). **B** and **C** illustrate the end-inspiratory and end-expiratory thickness, respectively, for patients divided according to the respiratory diagnosis (green, orange, violet and pink dots for patients without lung disease or with transient tachypnoea of the neonate, respiratory distress syndrome and neonatal acute respiratory distress syndrome, respectively). Box plots depict (from top to bottom) 95th, 75th, 50th, 25th, and 5th percentiles. The raincloud curves on the right side represent the density (distributions) of datapoints; for the color saturation effect, the overlaying curves may result in colors which do not correspond to the box and dot plots, but they visually describe the data overlap. Data were analyzed with Kruskal–Wallis test (overall *p* = 0.021 for both inspiratory and expiratory thickness); horizontal lines indicate significant post hoc comparisons (Conover-Iman test). Abbreviations: NARDS: neonatal acute respiratory distress syndrome; NLD: no lung disease; RDS: respiratory distress syndrome; TTN: transient tachypnoea of the neonate

### Electronic supplementary material

Below is the link to the electronic supplementary material.

Supplementary Video 1: Illustrative videoclip recorded on longitudinal scan and used for the pleural line thickness measurement.

Supplementary Video 2: Illustrative videoclip recorded on transversal scan and used for the pleural line thickness measurement.

## Data Availability

The datasets used and/or analyzed during the current study are available from the corresponding author on reasonable request that respect all relevant privacy and data property regulations.

## List of abbreviations

CV: Coefficient of variation
ICC: Intraclass correlation coefficient
NARDS: Neonatal acute respiratory distress syndrome
NICU: Neonatal intensive care unit
NLD: No lung disease
RDS: Respiratory distress syndrome
TTN: Transient tachypnoea of the neonate
